# Reimagining Medical Education Toward Antiracist Praxis

**DOI:** 10.1089/heq.2023.0135

**Published:** 2023-09-13

**Authors:** Russyan Mark Mabeza, Rupinder K. Legha

**Affiliations:** ^1^ Department of Surgery, University of California, San Francisco, San Francisco, California, USA.; ^2^ President, Rupinder K Legha, MD PC, Los Angeles, California, USA.

**Keywords:** antiracism, curriculum, medical education, critical race theory, abolition, decolonization

## Abstract

Medicine has a longstanding history of racism that promulgates existing health inequities. Current medical education, largely based on the biomedical framework, omits critical discourse on racism and White supremacy, which continue to harm individuals and communities of color. Such ahistorical and apolitical orientation inadequately trains learners to identify and address racism in clinical practice. Although curricula on racial health disparities, social determinants of health, cultural competency, and implicit bias have been operationalized by several medical schools, they do not identify the racism embedded in systems of care, nor do they provide transformative steps toward true health equity and justice. As such, this article proposes bold radical frameworks as the foundation for reimagining medical education in the United States. Founded on critical race theory, abolition, and decolonization, the authors provide a view of an antiracist medical education, one that highlights the history and legacy of racism in medicine and positions medical trainees and practicing physicians as active agents in medicine's antiracist transformation.

## Introduction

On February 22, 2022, Dr. Jeffrey Lieberman, chair of Columbia's Department of Psychiatry and a former president of the American Psychiatric Association, retweeted a photograph of a South Sudanese model referring to her as a “freak of nature.”^1^ On March 23, 2020, the University of Rochester Medical Center negligently discharged Daniel Prude from their emergency department, despite his family's pleas that he had attempted suicide while psychotic. Desperate for the help doctors had failed to provide, they called the police, who then killed Mr. Prude.^2^

On January 20, 2023, emergency medical services workers asphyxiated Earle Moore, Jr. to death by restraining him face down to a gurney after police called reporting a mental health emergency.^3^ These instances exemplify medicine's enduring and brutalizing legacy of racism, which persists, in part, because health care practitioners are not trained to identify nor address racism in clinical practice and the profession at large.

In 2020, we created the Antiracism in Medicine Curriculum Series (AMCS) to address the dearth of antiracist medical educational content that would prepare learners to avoid and prevent egregious acts of racism like these.^4^ Through AMCS, we sought to reimagine medical education into one that exposes the harms medicine has perpetrated against communities of color, potentiating its power as a tool for health equity. We moved beyond sanitized concepts that erase and evade discussion of the medical profession's role in perpetuating racist health inequities. In contrast, we challenged learners to be active agents in undoing this role through antiracist actions on structural, institutional, interpersonal, and intrapersonal levels. Such an activist orientation in education is necessary to shift the mindsets of the U.S. medical workforce and raise the next generation of antiracist physicians.

## Piloting Antiracist Medical Education Content

We piloted the “Antiracism in Mental Health” module at two different medical schools in 2022 and 2023. In the first pilot site, we partnered with a longitudinal child and adolescent psychiatry program, whose goal is to build a more inclusive workforce through mentorship, financial support, and various learning opportunities. In this program, we worked with 15 learners, whose clinical training ranged from first-year medical student to resident and fellow. The second pilot site was a 5-year medical education program whose mission is to raise up physician-leaders that will promote health equity, particularly for underserved communities in urban areas. This learning group involved 15 first-year medical students from diverse backgrounds, with close participation of program leadership and staff.

The module, which was facilitated over the course of 3 h, focused on the story of X, a 17-year-old nonbinary Mayan-Haitian biracial child who initially presented to the emergency room for suicidal ideation. Through X's narrative, interwoven with critical history and existing realities, we sought to decenter the dominant White heteronormative patriarchical gender-binary ableist narrative silencing the voices of people of color while recognizing the harm it has caused. The module underscores key racist inequities, like the overdiagnosis of schizophrenia^5^ as well as their derivation from histories of oppression,^6^ such as pathologizing of Black people,^7–9^ the carcerality of mental health “care,”^10^ and the eugenicist roots of race-based diagnostic tools and treatment guidelines.^11,12^

Furthermore, it guides medical trainees to recognize how White supremacy structures opportunity pertaining to housing and education to perpetuate communities of color's marginalization by design. The module disavows “color blindness,” instead fostering race consciousness and interrogating the intersecting axes of privilege and oppression shaping the care provided. We used these sessions as a vehicle to provide learners with the skills to interrogate upstream structures that enable downstream violence. During the pilot study, we pushed learners to fiercely scrutinize and undo carceral practices that put Black and brown patients in restraints and paint their responses to historical and present trauma as “combativeness” or “noncompliance.”

We emphasized valuing and revitalizing Indigenous knowledge and healing approaches as a means of disavowing scientific racism, White normativity, and White hegemony and dismantling their harm. The module highlights the medical profession's settler colonialist role, an awareness of which is necessary to move in solidarity with Indigenous communities. The final part of the module, which involved discussing antiracist action steps on structural, institutional, interpersonal, and interpersonal levels, led learners into critical reflection that positions them as antiracist agents, not mere observers of racial disparities with no role in dismantling them.

## Lessons Learned

This process gifted us with several insights pertaining to content and process that we hope will be helpful to coconspirators who hope to push forward an antiracist reimagining of medical education. Based on our experiences, we share some strategies for success in Table 1.

**Table 1.** Antiracism in Medicine Curriculum Series: Strategies for Success

Content		
	AMCS: Disruptive, transgressive medical education	“Business as Usual” approaches
Frameworks	CRT, abolition, and decolonization • Identify White supremacy and other intersecting systems of oppression's ubiquity • Unpack far-reaching harms pertaining to the carceral state • Cultivate an ongoing reflective practice and critical lens • Call for activism and an active undoing of histories of oppression, for example, through reparations.	Social determinants of health, cultural competency, and implicit bias • Render medical practitioners as inactive observers of poor outcomes experienced by minoritized patients • Simplify intersecting systems of oppression into a matter of effective cross-cultural interactions • Individualize racism to a matter of personal attitude and action, thereby obscuring the profession's responsibility to address multiple levels of racism.
Framing of current realities	Racist (health) inequities • Delineates how inequities pertaining to racism derive from far-reaching histories of oppression (such as slavery and colonization) • Emphasizes health inequities' interrelation with economic, educational, legal, and other inequities.	Health disparities • Describes “differences” pertaining to race as if they are static phenomena with no clear perpetrators/sources of harm or accountability • Points and stares at injustice instead of promoting justice.
Reckoning with history	Multiple arcs of racism • Segregation and structural racism: recognizes political, legal, and social agents of racist inequities • Racial essentialism and institutionalized racism: brings to light algorithms, guidelines, and clinical practices that perpetuate unjust care • Medical abuse and interpersonal racism: acknowledges forced experimentation and medical brutality • Medicine and the carceral state: highlights the intersection of medicine with police violence and oppressive justice system	Apolitical and ahistorical • Limitation within the biomedical framework • Insistence of medicine's “objectivity” • Promotes race as a biological notion rather than as a social construct • Upholds refusals of White supremacy by failing to confront injustice
Antiracist action	Multiple levels of antiracism • Structural • Institutional • Interpersonal • Intrapersonal	No antiracism • Fixates on the datafication of injustice • Describes what is already known, thereby condoning staying on the racist sidelines • Fails to enact accountability for well-established inequities • Lacks interdisciplinary approach
Approach		
	Positionality	Conflict of interest
	Creating a brave learning environment	No frame is set
	Meaningful dialogue	Silence/didactic approach
	Antiracism development • Rely on various developmental frameworks pertaining to racism (e.g., antiracism journey, racial identity development) to make sense of where learners and colleagues are at with racism, instead of relying on their racial phenotype.	Maintaining the status quo • Good intentions • “Something is better than nothing.” • Installing people of color in diversity, equity, and inclusion roles as a means of tokenization while maintaining the White supremacy culture of academic medicine
	Medical training as assimilation trauma	Medical training as acquisition of objective knowledge/expertise
Resources		
Personnel	White fragility acknowledged as a legitimate toxic exposure Solidarity building through relationship building across intersecting identities Leveraging of privilege while divesting from hierarchy and titles	White normativity Refusals of White supremacy Steep power dynamics Hierarchy of medicine Individualistic approach
Funding	Adequate financial compensation • Educators are provided appropriate compensation to ensure sustenance of antiracist efforts	Minority taxation and exploitation • Faculty/educators of color are frequently expected to educate majority White people about racism without adequate pay

AMCS, Antiracism in Medicine Curriculum Series; CRT, critical race theory.

First, the task begins by rejecting common concepts in medical education that maintain the status quo of racist health inequities, such as social determinants of health, cultural competency, and implicit bias. These concepts fail to name or condemn the White supremacy foundational to racism despite pro-White implicit bias's established role in shaping health outcomes. Encouraging static and descriptive approaches, they obscure histories of oppression and fail to cultivate the antiracist actions needed to challenge racism's far-reaching public health impact. Instead, our approach to pedagogy was founded on critical race theory (CRT), abolition, and decolonization, which are necessary frameworks to dissect racism and White supremacy's roles in medicine and strive to dismantle both.

Laying the foundation with these frameworks allowed us to develop a strategic approach for teaching antiracism. CRT provides the necessary conceptual foundation for antiracism in medical education because it analyzes race's social construction and racism's historical legacy to combat the root causes of structural violence.^13^ Abolition medicine is equally critical because it calls for constructing new systems of community-based care that challenge the medical–industrial complex rooted in slavery.^14^ Decolonization deconstructs colonial ideologies of the superiority and privilege of Western thought and approaches.^15^

Second, implementing bold and radical antiracist content in institutions rooted in White supremacy is often a hostile process, due to efforts being blocked left and right. It was very common for faculty and administrators to respond with questioning of our content, pushing back on our pedagogy, and expressing anxiety regarding backlash against the curriculum. Many times, logistical constraints squandered the possibility of teaching this content, be it scheduling, administrative approvals, or other forms of bureaucratic red tape.

In the two partner medical school programs where we have had success piloting this content, we were fortunate to have coconspirators who helped us navigate the political and bureaucratic mazes that would have prevented us from teaching this content altogether. As with any antiracism work, it is important to acknowledge the normalcy of such resistance placed by institutions. Doing so allows for development of a strategy that enables the antiracist work to materialize amid many possible obstructions.

To this point, we experienced firsthand the critical role of allyship. We witnessed partnering faculty and staff leverage their privilege, social capital, and institutional knowledge to ensure a successful piloting of the curriculum. These coconspirators helped us secure funding, physical (and virtual) space for delivering the curriculum, as well as close communication with all the participants. During sessions, they vulnerably shared their own positionalities and experiences, modeling the posture of learning that was necessary for transgressive learning.

Since they are more closely tied with the learners, they also partnered with us in caring for their well-being beyond the session, which included debriefing and sharing of feedback in smaller private settings. Their championship of our curricular effort and willingness to partner in its delivery were critical to its success.

Fourth, examining positionality is critical for facilitators and learners alike. In qualitative research, positionality has long been an integral element that defines the boundaries within which knowledge is produced and, therefore, interpreted.^16^ When discussing the history and legacy of racism in medicine, it is critical to interrogate our own histories and realities of oppression and domination, which shape the way we enter the shared learning space. Within AMCS, we applied Pettyjohn et al.'s discussion of intersectionality^17^ to examine the identities that form our biases, opinions, and relationship with the content.

Many learners experienced this part of the module as emotional and challenging, especially those who are in the beginning stages of their racial identity formation. For some, particularly those from marginalized backgrounds, the histories of oppression and medical abuse were not theoretical but rather lived. Interrogating each individual's positionality before diving into the content was essential in priming learners as active antiracist agents and promoting their well-being and safety throughout the process.

Fifth, discord and discontent can be seeds of solidarity, and must be normalized in discussions of medicine's racist past and present. Some of the most common feedback we have received throughout the sessions expressed disgust, frustration, and even helplessness. It was not infrequent for learners to question their choice of entering the medical profession, especially as they gained greater understanding of the structures and systems that continue to subjugate communities of color. We sat with these tensions and used them as opportunities to activate learners toward concrete action. Despite participants' varying intersectional identities, the shared discontent made way for solidarity, which advanced them from the fear to the learning, and to the growth zones of becoming antiracist.^18^

## Conclusion

The call to dismantle racism in medicine is present and urgent. To do so, medical training programs must make way for a bold and radical reimagining of medical education—one that rejects the status quo and interrogates the history of racism in medicine to detangle from its White supremacist legacy. Ibram Kendi's dichotomy of all actions and decisions being either racist or antiracist provides a necessary ultimatum: each one is either part of the problem or the solution. In humility and solidarity, it is up to each member of the medical profession, from first-year medical students to seasoned practicing physicians, to reimagine medical education to move medicine toward true antiracist healing.

## Abbreviations Used

AMCS: Antiracism in Medicine Curriculum Series
CRT: critical race theory
